# Optimization of ionizable lipids for aerosolizable mRNA lipid nanoparticles

**DOI:** 10.1002/btm2.10580

**Published:** 2023-08-21

**Authors:** Mae M. Lewis, Melissa R. Soto, Esther Y. Maier, Steven D. Wulfe, Sandy Bakheet, Hannah Obregon, Debadyuti Ghosh

**Affiliations:** ^1^ Department of Biomedical Engineering The University of Texas at Austin Austin Texas USA; ^2^ Division of Molecular Pharmaceutics and Drug Delivery, College of Pharmacy The University of Texas at Austin Austin Texas USA; ^3^ Drug Dynamics Institute The University of Texas at Austin Austin Texas USA

**Keywords:** aerosolization, ionizable lipid, lipid nanoparticle, mRNA, pulmonary delivery

## Abstract

Although mRNA lipid nanoparticles (LNPs) are highly effective as vaccines, their efficacy for pulmonary delivery has not yet fully been established. A major barrier to this therapeutic goal is their instability during aerosolization for local delivery. This imparts a shear force that degrades the mRNA cargo and therefore reduces cell transfection. In addition to remaining stable upon aerosolization, mRNA LNPs must also possess the aerodynamic properties to achieve deposition in clinically relevant areas of the lungs. We addressed these challenges by formulating mRNA LNPs with SM‐102, the clinically approved ionizable lipid in the Spikevax COVID‐19 vaccine. Our lead candidate, B‐1, had the highest mRNA expression in both a physiologically relevant air–liquid interface (ALI) human lung cell model and in healthy mice lungs upon aerosolization. Further, B‐1 showed selective transfection in vivo of lung epithelial cells compared to immune cells and endothelial cells. These results show that the formulation can target therapeutically relevant cells in pulmonary diseases such as cystic fibrosis. Morphological studies of B‐1 revealed differences in the surface structure compared to LNPs with lower transfection efficiency. Importantly, the formulation maintained critical aerodynamic properties in simulated human airways upon next generation impaction. Finally, structure–function analysis of SM‐102 revealed that small changes in the number of carbons can improve upon mRNA delivery in ALI human lung cells. Overall, our study expands the application of SM‐102 and its analogs to aerosolized pulmonary delivery and identifies a potent lead candidate for future therapeutically active mRNA therapies.

**Translational Impact Statement**

This study addresses challenges associated with delivering gene therapies locally to the lungs through inhalation. We identified a lipid nanoparticle formulation, B‐1, that achieves higher delivery of NLuc mRNA in a human lung cell model and in mouse lungs upon aerosolization compared to Onpattro, the first FDA‐approved lipid nanoparticle formulation for nucleic acid delivery. Importantly, aerosolized B‐1 also transfects the therapeutically relevant epithelial cell type within the lungs while maintaining promising aerodynamic properties in simulated human airways.

## 1 INTRODUCTION

Delivery of mRNA has a strong potential to treat and cure pulmonary genetic diseases such as cystic fibrosis (CF). For example, mRNA CRISPR‐Cas‐based therapies can directly edit the disease‐causing mutations on the cystic fibrosis transmembrane conductance regulator (CFTR) gene.^1^, ^2^, ^3^ Although there are over 2000 mutations that cause CF, the versatility of mRNA allows it to be easily tailored for individual patients.^4^ mRNA can also encode for native CFTR that would treat CF patients regardless of their mutation type.^5^, ^6^ While mRNA is readily tunable for both purposes, it is inherently unstable and degrades rapidly in vivo.^7^, ^8^

Lipid nanoparticles (LNPs) are attractive carriers that can successfully encapsulate mRNA and enhance its intracellular expression.^9^, ^10^, ^11^, ^12^, ^13^, ^14^, ^15^, ^16^ Most remarkably, the development of mRNA LNPs accelerated vaccine formulation at an unprecedented pace to alleviate the COVID‐19 pandemic.^17^, ^18^ As a testament to its versatility, the platform has strong investment by many companies in ongoing Phase I Clinical trials to deliver mRNA for other viral infections, autoimmune disorders, cancers, and genetic diseases.^19^ Despite their advances in other applications, there has only been one completed clinical trial for the delivery of mRNA LNPs to the lungs.^20^ The Phase 1/2 clinical trial for CF patients (RESTORE‐CF) evaluating an LNP encapsulating CFTR mRNA showed safety and tolerability after repeated dosing but did not significantly improve lung function. Therefore, there is still a need for a translatable mRNA LNP for pulmonary delivery.

The chemistry of the lipid components as well as their ratios are critical factors in identifying lead LNPs for other applications.^21^, ^22^ An LNP generally consists of four components: (1) a phospholipid (helper lipid) which influences LNP structure and enhances endosomal escape of mRNA, (2) cholesterol which tunes LNP flexibility and stability, (3) a PEG‐lipid which modulates LNP size and inhibits LNP uptake by immune cells,^23^, ^24^ and (4) an ionizable lipid which has a tertiary amine that becomes positively charged at a low pH to allow for mRNA encapsulation during formulation and endosomal escape after delivery.^25^, ^26^, ^27^ As ionizable lipids are critical in the complexation and release of nucleic acid cargo, efforts to improve delivery have mainly focused on modifying this component. Large screens exploring lipid chemistry have resulted in three potent ionizable lipids used in FDA‐approved products: DLin‐MC3‐DMA (MC3) used in the patisiran (Onpattro) siRNA LNP treating transthyretin‐mediated amyloidosis,^28^, ^29^ SM‐102 used in the mRNA‐1273 (Spikevax) mRNA LNP vaccine for COVID‐19,^12^, ^30^ and ALC‐0315 used in the BNT162b2 (Comirnaty) mRNA LNP vaccine for COVID‐19.^30^ As these three lipids have demonstrated potency and efficacy, they represent ideal starting points to improve upon LNP delivery to the lungs while aiding in rapid clinical translation.

The efficacy of the LNPs is also highly dependent on the route of administration. In harnessing LNPs for pulmonary delivery, there are two methods of administration: systemic and locally inhaled. Extensive work has shown that upon systemic delivery, many LNP formulations have had tropism for the liver due to the binding of circulating ApoE protein, which subsequently trafficked the particles to the organ.^28^, ^29^ To address this, a shift in tropism from the liver to the lung has been accomplished with the selective organ targeting strategy whereby a permanently cationic excipient is added to the existing four‐component system^31^ and with N‐series LNPs that have chemically modified lipidoids.^32^ While these compositions have improved delivery into the lung, inhaled therapies remain a promising avenue that offer increased concentration to the site of action, more facile delivery to airway epithelia, less off‐target effects compared to systemic delivery, and better patient compliance.^33^, ^34^, ^35^

To make an inhalable medicine, the drug must be effectively aerosolized. While aerosolization has been successful for an FDA‐approved liposomal formulation encapsulating an antibiotic,^36^ the shear force causes degradation of LNPs encapsulating nucleic acid and therefore subsequent loss of delivery.^37^, ^38^ The formulation must be rigid enough to withstand this force while also having the ability to undergo membrane fusion for cell uptake and endosomal escape. Finally, the LNP must achieve delivery at clinically relevant locations along the respiratory tract. For example, CF requires a therapy that deposits in the epithelial cells throughout the lungs in both the small and large airways.^39^, ^40^ An ideal LNP for pulmonary delivery would address all of these barriers. While the effect of lipid type and component ratios on potency and stability have been explored for aerosolized mRNA LNPs,^41^, ^42^, ^43^, ^44^ these studies have not focused on human airway deposition. To address this gap, we built upon our previous work on aerosolized mRNA LNPs^45^ by screening varying component ratios and all three clinically approved ionizable lipids to identify lead candidates and test their ability as aerosolized formulations to transfect airway epithelia and deposit throughout simulated airways and in vivo.

## 2 RESULTS

### 2.1 Delivery of mRNA LNPs to human lung cells cultured at air–liquid interface

**FIGURE 1:**
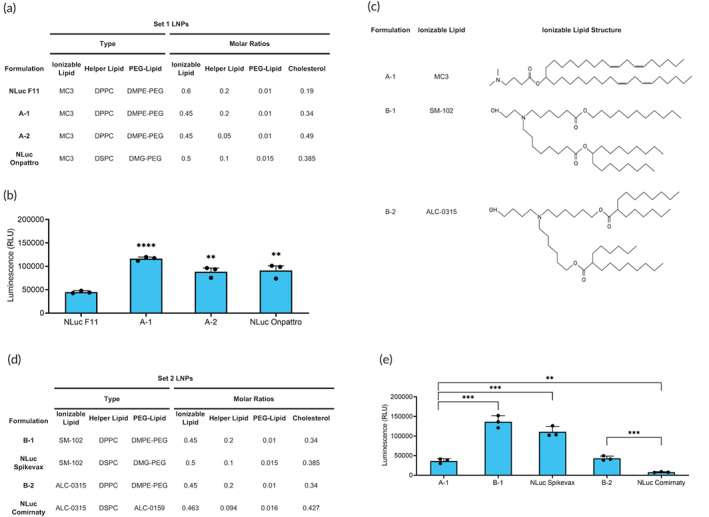
In vitro delivery in ALI Calu‐3 cells. (a) Compositions of set 1 LNPs. (b) Quantification of luminescence from ALI Calu‐3 cells 24 h after transfection with aerosolized set 1 LNPs delivering 1000 ng NLuc mRNA. Significance is relative to NLuc F11 (*n* = 3; mean ± standard deviation [SD], **p < 0.01, ***p < 0.001; Student's *t* test, double‐tailed). (c) Structures of ionizable lipids used in sets 1 and 2 LNPs. (d) Compositions of set 2 LNPs. (e) Quantification of luminescence from ALI Calu‐3 cells 24 h after transfection with aerosolized set 2 and A‐1 LNPs delivering 1000 ng NLuc mRNA (*n* = 3; mean ± SD, **p < 0.01, ***p < 0.001; Student's *t* test, double‐tailed). ALI, air–liquid interface; LNP, lipid nanoparticle.

We designed the first set of LNPs based on four‐component compositions that successfully delivered firefly luciferase (FLuc) mRNA to lung cells after aerosolization.^45^ Building upon this work, we screened for more potent component ratios and ionizable lipids based on F11, the LNP which showed the highest delivery in vivo. F11 was formulated with MC3, DPPC, DMPE‐PEG, and cholesterol at molar ratios 0.6:0.2:0.01:0.19. From this, we formulated a first set (set 1) of LNPs that varied the MC3, DPPC, and cholesterol component ratios while keeping the PEG‐lipid constant at 0.01 (Figure 1a). In addition, set 1 LNPs encapsulated nanoluciferase (NLuc) mRNA, a reporter molecule that is ~100× more sensitive than FLuc. We also included the F11 and Onpattro formulations encapsulating NLuc mRNA (NLuc F11 and NLuc Onpattro, respectively) as controls. We then aerosolized the LNPs by vibrating mesh nebulizer and assessed transfection efficiency in human bronchial epithelial Calu‐3 cells cultured at air–liquid interface (ALI), which is a more physiologically relevant culture method to better mimic the lung airspace compared to submerged plated cells.^46^ At ALI, Calu‐3 cells are an established model for screening inhalable drugs as they acquire in vivo‐like barrier properties such as formation of tight junctions and secretion of mucus components.^47^, ^48^, ^49^, ^50^ To quantify tight junction integrity, we measured transepithelial electrical resistance (TEER). After being cultured at ALI for 1 week, Calu‐3 cells demonstrated intact tight junctions with TEER values >400 Ω*cm^2^. Each LNP was delivered at a dose of 1000 ng to the apical side. Twenty‐four hours post‐administration, we harvested the cells and measured luminescence intensity. Decreasing the MC3 ratio from 0.6 to 0.45 while increasing the cholesterol ratio resulted in significantly higher luminescence for both A‐1 and A‐2 compared to NLuc F11 (Figure 1b). The best performing formulation, A‐1, had a 2.6‐fold increase compared to NLuc F11 and a 1.3‐fold increase compared to NLuc Onpattro. Therefore, this formulation served as the basis for the second set of LNPs (set 2).

We formulated set 2 LNPs to test the clinically approved ionizable lipids and compare their impact on transfection. While the structures of SM‐102 and ALC‐0315 differ from MC3 (Figure 1c), the component ratios of the vaccines are similar to that of A‐1.^30^ The similarity makes SM‐102 and ALC‐0315 compatible with our screening method without having to alter the base formulation to test their efficacy. We therefore formulated set 2 LNPs to test SM‐102 (B‐1) and ALC‐0315 (B‐2) while holding the component ratios, helper lipid type, PEG‐lipid type, and cholesterol type constant from A‐1 (Figure 1d). We also made formulations that consisted of the same components in the COVID‐19 vaccines Spikevax (SM‐102:DSPC:DMG‐PEG 2000:cholesterol 0.50:0.10:0.015:0.385) and Comirnaty (ALC‐0315:DSPC:ALC‐0159:cholesterol 0.463:0.094:0.016:0.427) as controls for B‐1 and B‐2, respectively.^30^ As these LNPs encapsulate NLuc mRNA rather than their original therapeutic payloads, we named the formulations NLuc Spikevax and NLuc Comirnaty. Set 2 LNPs were screened similarly to set 1 LNPs. B‐1 exhibited the highest transfection efficacy, with a 3.8‐fold increase in luminescence compared to A‐1, while B‐2 was significantly lower (Figure 1e). In addition, both B‐1 and B‐2 LNP formulations had higher luminescence compared to their vaccine counterparts, although this increase was not statistically significant for B‐1. Taken together, both screens identify a lead candidate, B‐1, while highlighting the importance of ionizable lipids in enhancing mRNA delivery to ALI Calu‐3 cells.

To investigate whether an improved transfection before aerosolization correlated with improved transfection after aerosolization, we compared nonaerosolized and aerosolized sets 1 and 2 LNPs (Figure S1). There were two different observations depending on the level of transfection. We found that particles with lower transfection before aerosolization had lower transfection after aerosolization. For example, NLuc F11 had decreased luciferase expression both before and after aerosolization compared to A‐1. However, this was not the case for particles with higher transfection. Both B‐1 and NLuc Spikevax had significantly higher luciferase expression after aerosolization compared to A‐1 despite all three LNPs having similarly high luciferase expression before aerosolization. In our previous work, we observed a similar trend: some formulations have a large decrease in delivery after aerosolization despite being identified as lead candidates before aerosolization.^45^ Overall, these data show an improved transfection before aerosolization does not necessarily ensure improved transfection after aerosolization.

Interestingly, the qualities that allow the LNP to better withstand aerosolization were not explained by physicochemical characterization. LNPs from both sets had similar changes in size, PDI, zeta, and encapsulation efficiency. Before aerosolization, all LNPs were stable with a size range below 125 nm, a polydispersity index (PDI) range below 0.2, and a zeta potential range from −1.7 to −10.3 mV measured by Dynamic light scattering (DLS; Figure S2a–c). All LNPs also had high mRNA encapsulation efficiency (87.2%–93.9%; Figure S2d). After aerosolization, all LNPs experienced an increase in size and PDI as well as a decrease in zeta potential. In addition, the encapsulation efficiency decreased. These properties have been noted by other mRNA LNP studies using mesh nebulizers.^42^, ^43^ As none of these properties correlate with transfection efficiency, screening early for delivery after aerosolization was important for identifying a lead candidate.

### 2.2 Delivery of NLuc mRNA LNPs to mouse lungs

**FIGURE 2:**
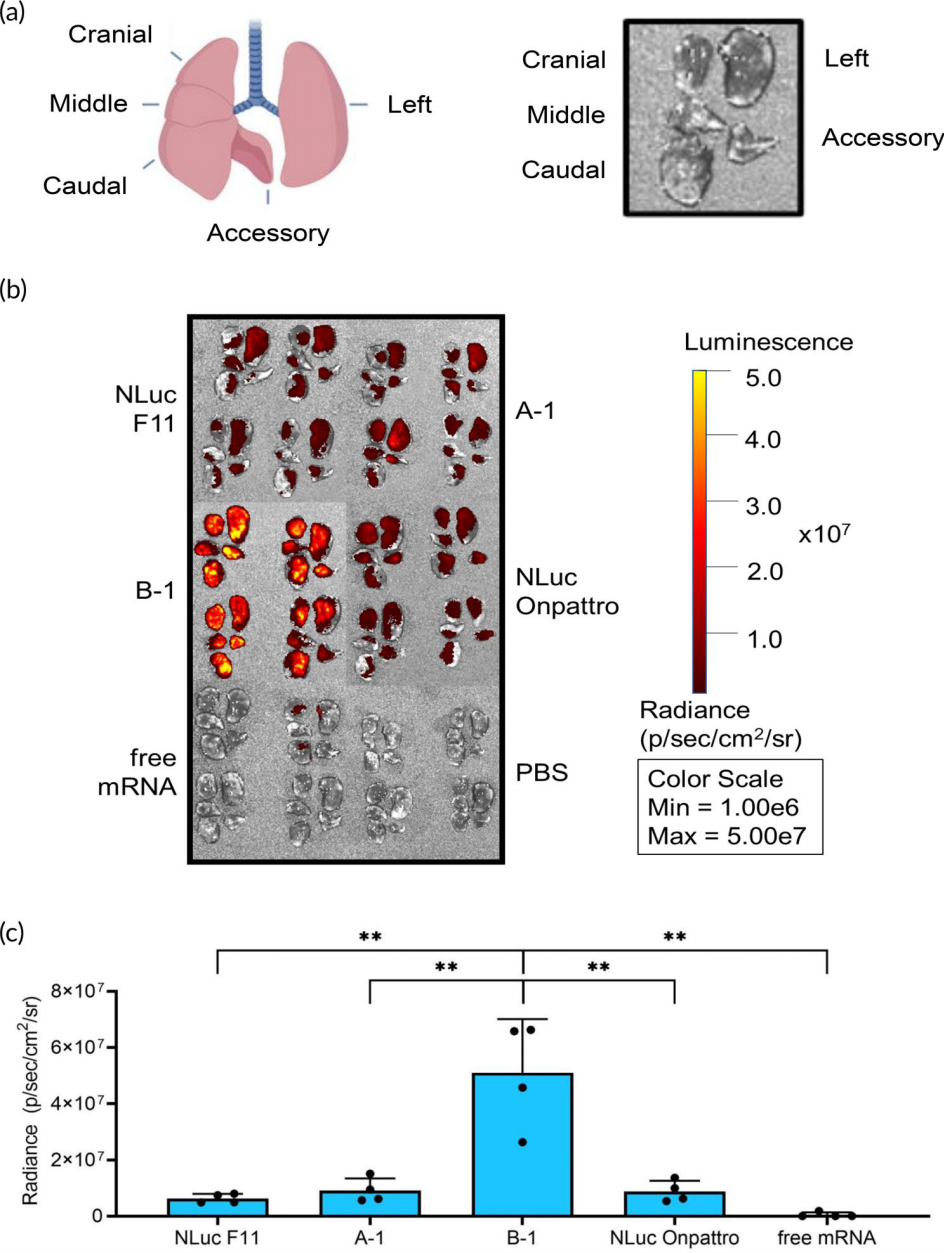
In vivo delivery in mouse lungs. (a) Diagram of five mouse lung lobes in vivo and after harvesting. Created with BioRender.com. (b) Radiance in the five individual BALB/c mice lung lobes 24 h after intratracheal administration of aerosolized LNPs delivering 750 ng NLuc mRNA. Parameters were kept constant throughout imaging. (c) Quantification of radiance from Figure 2b (*n* = 4; mean ± standard deviation, **p < 0.01, ***p < 0.001; Student's *t* test, double‐tailed). LNP, lipid nanoparticle.

For diseases that affect the whole airway, a successfully inhaled mRNA LNP must have high transfection efficiency throughout the lungs. Here, we tested the transfection efficiency of aerosolized NLuc mRNA LNPs in the lungs of healthy mice. We delivered either NLuc F11, A‐1, B‐1, or NLuc Onpattro to BALB/c mice through intratracheal administration after aerosolization by vibrating mesh nebulizer and compared to an unencapsulated NLuc mRNA control (i.e., free mRNA). Twenty‐four hours post‐administration, mice lungs were harvested and separated into their five individual lobes (left, cranial, middle, caudal, and accessory). Lobes were prepared and imaged to quantify luminescence (Figure 2a).

All formulations had similar distributions throughout each of the five lobes. Additionally, all LNPs achieved higher mRNA expression compared to free mRNA throughout all lung lobes (Figure S3). Overall, B‐1 exhibited the highest radiance. The transfection efficiency of B‐1 was 8.1×, 5.6×, 5.8×, and 103.2× higher than NLuc F11, A‐1, NLuc Onpattro, and free mRNA, respectively (Figure 2b,c). Although A‐1 and NLuc Onpattro exhibited increased transfection compared to NLuc F11, the difference was not statistically significant. Therefore, B‐1 was selected as the lead candidate for its delivery efficacy in both ALI Calu‐3 cells and healthy mouse lungs.

### 2.3 Assessment of cell types transfected by B‐1 in the lungs

**FIGURE 3:**
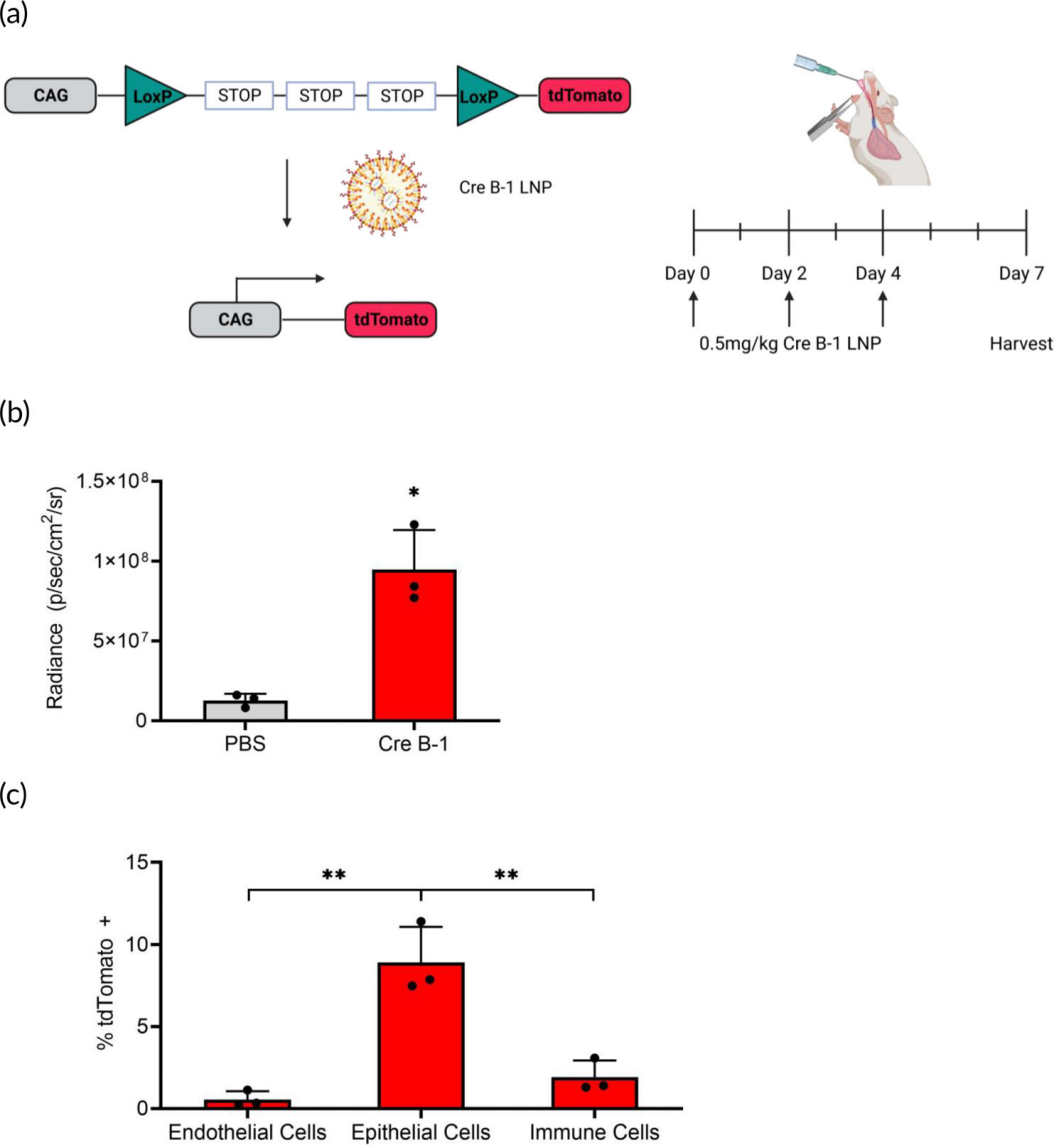
Assessment of cell types in the lungs for in vivo delivery. (a) Schematic of Rosa26 locus in Ai9 mice and delivery schedule of Cre B‐1 LNP. Upon successful Cre‐mediated recombination, the stop cassette will be deleted, and the CAG promoter will drive expression of tdTomato. Aerosolized Cre B‐1 LNP was delivered at 0.5 mg/kg once every other day over a period of 4 days. Lungs were harvested 3 days after the last dose. Created with BioRender.com. (b) Quantification of tdTomato radiance from Ai9 mouse lungs after delivery of Cre B‐1 compared to PBS control. (c) Assessment of tdTomato‐positive cells in endothelial, epithelial, and immune cell populations using flow cytometry (*n* = 3; mean ± standard deviation, **p < 0.01; Student's *t* test, double‐tailed). LNP, lipid nanoparticle.

To track delivery and quantify the ability of B‐1 to transfect therapeutically relevant cell types within the lungs after aerosolization, we utilized the Ai9 reporter mouse model (Figure 3a).^51^ These mice contain a stop cassette flanked by LoxP sites in the Rosa26 locus preventing transcription of a fluorescent tdTomato protein. Upon Cre enzyme‐mediated recombination of the LoxP sites, the stop cassette will be deleted and tdTomato will be expressed. We can subsequently isolate tdTomato‐positive cells in conjunction with select surface markers via flow cytometry to identify which cell type our formulation is transfecting.

We delivered our B‐1 LNP encapsulating Cre mRNA and harvested the lungs after 7 days for analysis (Figure 3a). Through fluorescent imaging, B‐1 had significantly higher radiance than the phosphate‐buffered saline (PBS) control (Figure 3b). Through flow cytometry, we found that 8.9% of the epithelial cells, 1.9% of the immune cells, and 0.6% of the endothelial cells were tdTomato‐positive (Figure 3c). Upon local delivery, there was greater relative uptake in epithelial cells compared to other cell types, which is important in CF where the epithelia are the primary target for gene editing therapies. It has been shown that approximately 5%–50% of epithelial cells need to express functional CFTR to improve lung function in CF patients.^40^ These findings indicate that the cell transfection properties of aerosolized B‐1 are promising.

B‐1 targets relatively more of the desired epithelial cells compared to other lung‐targeted LNPs.^31^ Compared to an intravenously delivered five‐component LNP with tropism for the lung, B‐1 had higher relative uptake in epithelial cells compared to immune cells (4.7‐fold vs. 1.9‐fold). Additionally, the transfection of B‐1 in endothelial cells is significantly lower (0.6% vs. 66%). Here, the route of administration can play a role in delivery. Our LNP formulation is intended for local, pulmonary delivery to reach the epithelia, whereas the other formulation is delivered intravenously and has to overcome the vascular endothelium before it can access the epithelia. This comparison highlights the advantages of local delivery for LNP uptake in lung epithelial cells.

### 2.4 Characterization of LNP morphology before and after aerosolization

**FIGURE 4:**
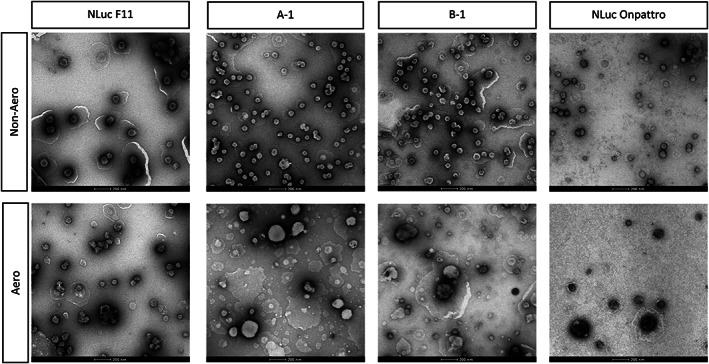
TEM images before and after aerosolization. TEM, transmission electron microscopy.

We used transmission electron microscopy (TEM) to evaluate the morphology of NLuc F11, A‐1, B‐1, and NLuc Onpattro. Before aerosolization, all formulations have a spherical structure as seen in other TEM and cryo‐TEM images of mRNA LNPs (Figure 4).^42^, ^52^, ^53^ After aerosolization, formulations have rougher spherical shapes with a larger size distribution as confirmed by the DLS data (Figure S2a,b). However, there are differences between B‐1 and the LNPs formulated with MC3. While all LNP samples contain particles that are larger than their nonaerosolized counterparts, these particles in B‐1 have more defects on the surface. Previously, imperfections in the lipid surface have been hypothesized to be linked to lipid type and subsequently transfection efficiency of mRNA LNPs.^54^ Therefore, these differences may suggest the importance of ionizable lipid type for aerosolized mRNA LNPs and show that the most morphologically intact particle may not have the highest transfection efficiency.

### 2.5 Next‐generation impaction to evaluate aerodynamic properties of LNPs

**FIGURE 5:**
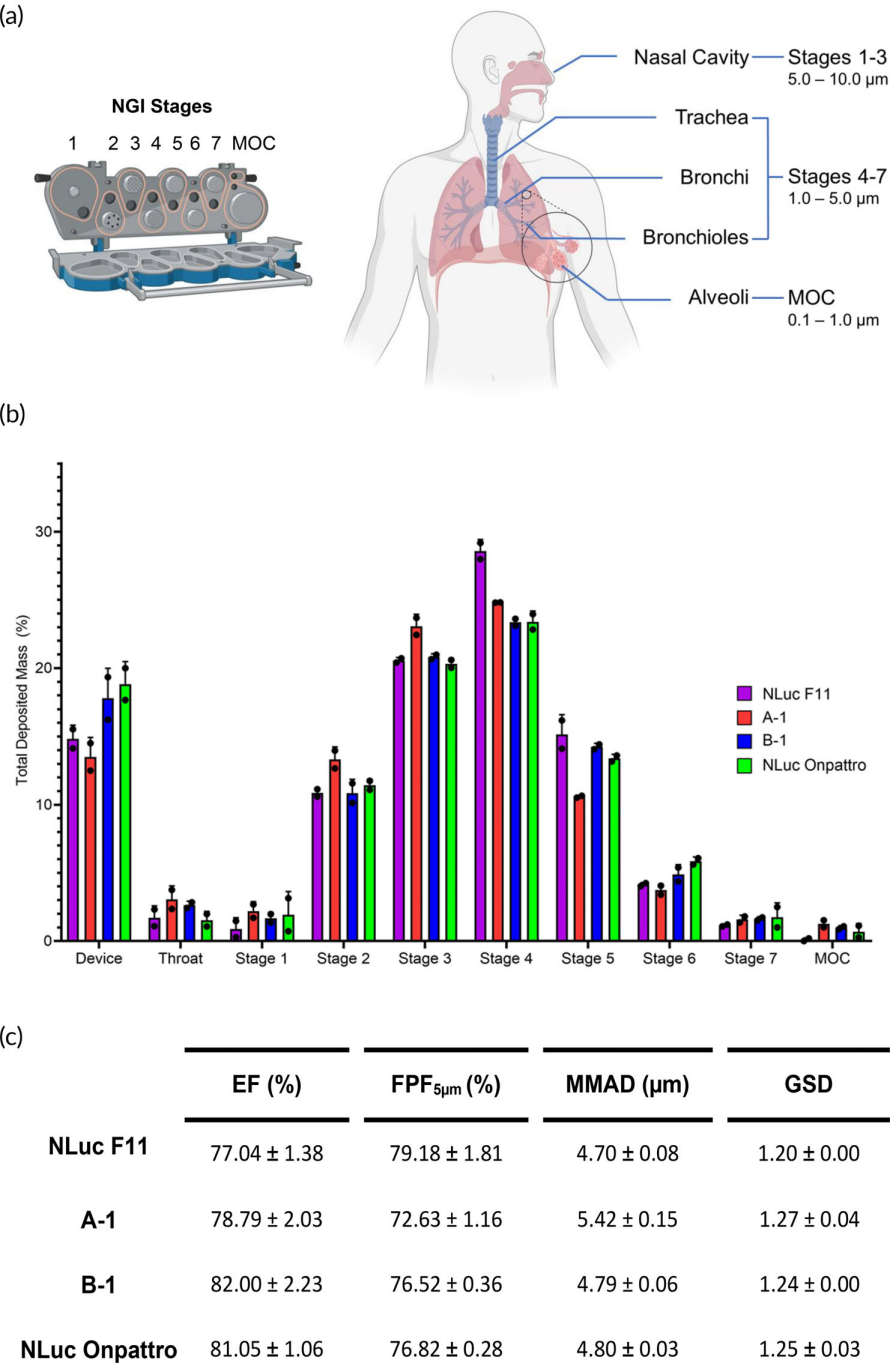
NGI at a flow rate of 15 L/min. (a) NGI stages and droplet distribution in the human respiratory tract according to particle size. Created with BioRender.com. (b) Deposition profile across the device, throat, stages 1–7, and MOC (*n* = 2; mean ± standard deviation [SD]). (c) Aerodynamic performance (*n* = 2; mean ± SD). MOC, micro‐orifice collector; NGI, Next Generation Impactor.

The structure of the lung anatomy is complex and branched. Ideally, an inhaled therapy as an aerosol must have droplet sizes ranging from 1 to 5 μm to avoid being expired (i.e., breathed out after inhalation) or caught in the upper airways.^55^, ^56^, ^57^ To evaluate the aerosol distribution of NLuc F11, A‐1, B‐1, and NLuc Onpattro in human lungs, we used a Next Generation Impactor (NGI). This device uses an airflow to allow aerosolized formulations to deposit along a throat, seven stages, and a micro‐orifice collector (MOC). The airflow rate through the NGI determines the aerodynamic diameter of the particles in each component and therefore their distribution within the lungs. At a flow rate of 15 L/min, particles collected from stages 1 to 3 deposit in the nasal cavity, while particles collected from stages 4 to 7 and the MOC deposit throughout the therapeutically relevant areas of the lung (Figure 5a).^58^ After each run, LNPs were collected from the NGI components and quantified with the RiboGreen assay. These values were used to calculate the emitted fraction (EF), fine particle fraction <5 μm (FPF_5μm_), median mass aerodynamic diameter (MMAD), and geometric standard deviation (GSD).^59^ The EF is the percentage of all particles deposited in the NGI components that are recovered from the throat, stages 1–7, and MOC. The FPF_5μm_ is the percentage of emitted particles that have diameters <5 μm. The MMAD is the diameter at which 50% of the aerosol droplets are larger or smaller with a benchmark value being the limit of settling in the lower airways (5 μm). The GSD represents the spread of the aerosol particle size distribution. A majority of A‐1, B‐1, NLuc F11, and NLuc Onpattro controls that deposited within the NGI components were collected from stages 4 to 7 and the MOC, which indicates the potential for deposition throughout the small and large airways (Figure 5b). This correlates with the high EFs (77.04%–82.00%; Figure 5c). Importantly, the FPF_5μm_ range of 72.63%–79.18% highlights that a majority of the particles are within the respirable range. In comparison, this fraction is greater than that of Arikayce (FPF_5μm_: 50.3%–53.5%), the only clinically approved liposomal drug developed for inhalation.^60^, ^61^ Compared to Arikayce, the MMAD is also <5 μm for all LNPs except A‐1. In addition, the GSD was also less than 2 for all LNPs.

### 2.6 Structure–function analysis on SM‐102

**FIGURE 6:**
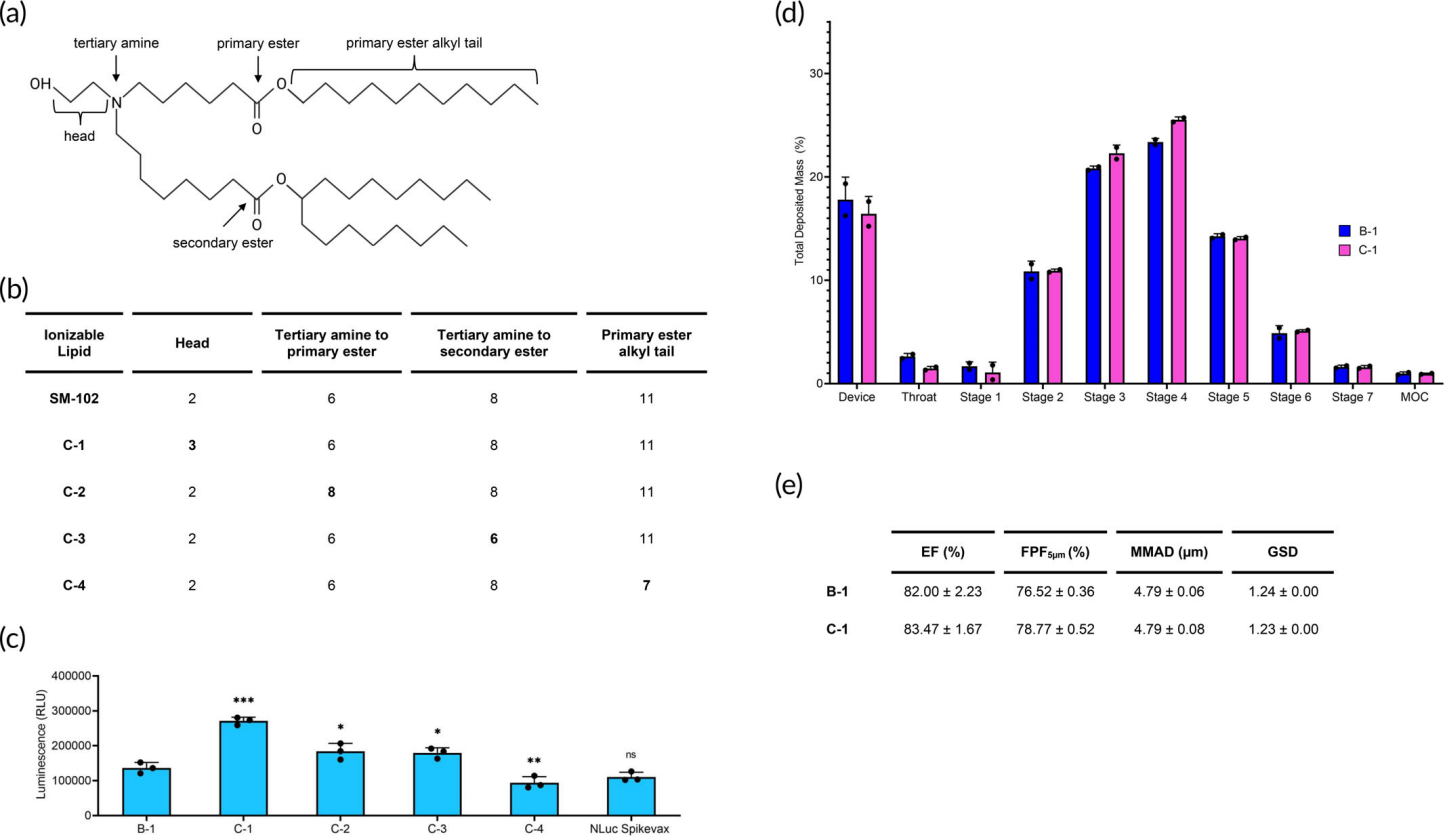
The impact of SM‐102 analogs on LNP transfection. (a) Schematic of SM‐102 groups relevant to the analogs. (b) The number of carbons in SM‐102 analog structures compared to SM‐102. (c) Quantification of luminescence from ALI Calu‐3 cells 24 h after transfection with aerosolized SM‐102 analog LNPs delivering 1000 ng NLuc mRNA. Significance is relative to B‐1 (*n* = 3; mean ± standard deviation [SD], *p < 0.05, **p < 0.01, ***p < 0.001, ns = not statistically significant; Student's *t* test, double‐tailed). (d) Deposition profile of C‐1 compared to B‐1 across the device, throat, stages 1–7, and MOC (*n* = 2; mean ± SD). (e) Aerodynamic performance of C‐1 compared to B‐1 (*n* = 2; mean ± SD). ALI, air‐liquid interface; LNP, lipid nanoparticle; MOC, micro‐orifice collector.

From these findings, we hypothesized that modifying the structure of the SM‐102 ionizable lipid would allow us to screen for analogs that improve transfection in airway epithelia. Based on another screen of other mRNA LNPs formulated with SM‐102 analogs, the alcohol head group, two esters, and branched tails are critical for transfection efficiency.^62^ Therefore, we retained these properties and instead focused on small changes in other carbon structures. We screened four analogs with changes to the number of carbons in the head, between the tertiary amine and both the primary and secondary esters, and the alkyl tail off of the primary ester (Figure 6a,b). Each of the analogs was used to formulate LNPs, and these LNPs were compared to B‐1 while maintaining the A‐1 component ratios. NLuc Spikevax was formulated as a control. Twenty‐four hours after delivering aerosolized LNPs to ALI Calu‐3 cells, we harvested the cells and measured luminescence.

We observed three analogs with significantly higher luciferase expression compared to SM‐102 (B‐1) (Figure 6c). C‐1, which has a 3‐carbon head, produced the highest increase by 2.0‐fold. C‐2 and C‐3, which both modify the position of the esters from the tertiary amine, increased luminescence by 1.3‐fold each. Another study also reported increased luciferase expression by formulating mRNA LNPs with carbon analogs. In Hassett et al.,^12^ lipids M, P, and Q increased FLuc expression after intramuscular delivery compared to SM‐102. Although these analogs are not exact matches of C1–4, shared features between this study and our study include a three‐carbon head and eight carbons between the tertiary amine and primary ester. Overall, we identified more potent ionizable lipids and can confirm that their chemistry impacts function for better delivery in ALI Calu‐3 cells, but further studies are warranted to elucidate the structure–activity relationship.

Similarly to B‐1, a majority of C‐1 LNPs deposit within stages 4–7 and the MOC (Figure 6d). Additionally, C‐1 has a high EF (83.47 ± 1.67%) and FPF_5μm_ (78.77 ± 0.52%) as well as an MMAD below <5 μm (4.79 ± 0.08 μm) and a GSD below 2 (1.23 ± 0.00; Figure 6e). While the chemistry can improve the transfection efficiency for SM‐102 analogs, this does not impact the potential for deposition in the human lungs.

## 3 DISCUSSION

LNPs have cemented their relevance for mRNA delivery in modern medicine through the transformative mRNA COVID‐19 vaccines.^17^, ^18^ This carrier can be widely used for many indications and more specifically has the potential to treat pulmonary genetic disorders through aerosolization. Although aerosolized mRNA LNPs have entered clinical trials for the treatment of CF^20^, the carrier needs to satisfy design criteria to be suitable for inhaled delivery. In this study, we improved upon carrier design by screening for LNPs that addressed two challenges: (1) degradation of the particle during aerosolization and (2) particle deposition in therapeutically relevant locations. Our lead SM‐102 LNP, B‐1, was stable upon aerosolization and achieved the highest transfection efficiency in ALI human lung cells and in healthy mice lungs. Further, Cre B‐1 had an increased transfection efficiency in epithelial cells (8.9%) compared to immune cells (1.9%) or endothelial cells (0.6%) in the lung. The high transfection rate of aerosolized Cre B‐1 in epithelial cells is promising for pulmonary diseases like CF.^63^ In addition, B‐1 also had the desired droplet properties in simulated human airways. Through NGI, B‐1 exhibited an FPF_5μm_ higher than Arikayce, the only clinically approved liposomal drug for inhalation with a mesh nebulizer.^60^, ^61^ Importantly, a high FPF_5μm_ translates to a greater percentage of therapeutically active mRNA. While transfection experiments and NGI assess different drug characteristics, each method had valuable criteria for an aerosolized formulation. Transfection shows the ability of the cell to translate and express the protein of interest after mRNA delivery, while NGI shows the potential of the formulation to deposit in clinically relevant locations throughout simulated human airways. A therapeutically relevant LNP must be able to do both. Because SM‐102 showed the most promising transfection efficiency while maintaining critical aerosol properties, we further improved upon the structure by screening for analogs. Our data identified a more potent LNP formulated with C‐1 which had three carbons in the head structure instead of two. This provides a starting point for synthesizing new ionizable lipids for pulmonary delivery.

From our findings, we would like to highlight possible routes of exploration for future work. In this study, we observed delivery and uptake throughout the lungs and in the epithelial cells with our select LNPs (Figures 2b,c and 3b,c). While we report that morphology can be implicated in transfection, this work is not focused on studying the effect of LNP structure on the endosomal escape of SM‐102. Other studies have noted that LNP surface morphology correlates with endosomal escape and is therefore important for transfection efficiency.^54^, ^64^ As a starting point, our study revealed morphology differences between B‐1 and LNPs formulated with MC3 (NLuc F11, A‐1, and NLuc Onpattro) upon aerosolization (Figure 4). Because this property is only present on the aerosolized formulations, we speculate that lipid rearrangement and LNP fusion due to shear force imparted by nebulization is a contributing factor. More specifically, we hypothesize that the four double‐bonds in MC3 may be responsible for limiting the flexibility of the A‐1 upon aerosolization, whereas the less constrained SM‐102, with no double bonds, may allow B‐1 to be more susceptible to structural changes. However, further studies are warranted to elucidate whether or not these differences affect endosomal escape. Finally, we did not address the mechanism behind the enhanced delivery of select SM‐102 analogs. Future screens could expand the type of analogs tested to elucidate the relationship between structure–activity and transfection efficiency by potentially varying the number of carbons or modifying the ester and hydroxy groups.

## 4 CONCLUSIONS

In this study, we established select LNP candidates that possess desired physicochemical properties for stable encapsulation and aerosolization, mRNA delivery for suitable expression in human cells and animal airways, and deposition in simulated human airways. In particular, we show that formulations with SM‐102 have superior transfection compared to compositions with other ionizable lipids. While further studies are warranted to improve performance, this work underscores the promise that LNPs can be aerosolized for delivery of an mRNA nucleic acid therapy.

## 5 MATERIALS AND METHODS

### 5.1 In vitro transcription

NLuc mRNA was codon‐optimized using the GenSmart™ Codon Optimization online tool through GenScript. Then, a template for NLuc mRNA was created by PCR‐amplifying a custom gene block ordered from Twist Biosciences encoding sequences for a T7 promoter, 5′ UTR, codon‐optimized NLuc, and 3′ UTR.^65^ All mRNA was synthesized and purified as described previously.^21^, ^66^ Briefly, the amplicon was transcribed using AmpliScribe™ T7‐Flash Transcription Kit (Lucigen, ASF‐3507) following manufacturer instructions. Following transcription, mRNA was purified using RNA Clean & Concentrator‐100 (Zymo, R1019). After purification, the cap1 structure was added using the Vaccinia Capping System (NEB, M2080S) and mRNA Cap 2′‐O‐methyltransferase (NEB, M0366S). A 3′‐poly(A) tail approximately 100 bp long was then added enzymatically using *E. Coli* Poly (A) Polymerase (NEB, M0276L). After polyadenylation, mRNA was purified again using RNA Clean & Concentrator‐100. mRNA concentration was determined by Nanodrop 1000 (Thermo Fisher Scientific Inc.), and aliquots were stored at −80°C until use.

### 5.2 Lipids

The ionizable lipid MC3 ((6Z,9Z,28Z,31Z)‐heptatriacont‐6,9,28,31‐tetraene‐19‐yl 4‐(dimethylamino)butanoate) was purchased from BioFine International Inc. The lipids SM‐102 (Heptadecan‐9‐yl 8‐{(2‐hydroxyethyl)[6‐oxo‐6‐(undecyloxy)hexyl]amino}octanoate), C‐1 (BP‐26399), C‐2 (BP‐26367), C‐3 (BP‐26361), C‐4 (BP‐26371), ALC‐0135 ([(4‐Hydroxybutyl)azanediyl]di(hexane‐6,1‐diyl) bis(2‐hexyldecanoate)), and ALC‐0159 (2‐[(polyethylene glycol)‐2000]‐N,N‐ditetradecylacetamide) were purchased from BroadPharm. The helper lipids DPPC (1,2‐dipalmitoyl‐sn‐glycero‐3‐phosphocholine) and DSPC (1,2‐distearoyl‐sn‐glycero‐3‐phosphocholine) were purchased from Avanti Polar Lipids. The PEG‐lipids DMG‐PEG 2000 (1,2‐dimyristoyl‐rac‐glycero‐3‐methoxypolyethylene glycol‐2000) and DMPE‐PEG 2000 (1,2‐dimyristoyl‐sn‐glycero‐3‐phosphoethanolamine‐N‐[methoxy(polyethylene glycol)‐2000]) were purchased from NOF America Corporation. Cholesterol was purchased from Sigma‐Aldrich.

### 5.3 LNP formulation

LNPs were formulated using a microfluidic setup described previously.^45^ Briefly, aliquots of each lipid at 10 mg/mL in 100% ethanol were combined at the appropriate molar ratios. The NLuc mRNA was diluted in 50 mM sodium citrate buffer with pH = 3.0. The organic and aqueous phases were combined at a 3:1 ratio with a total flow rate of 9 mL per minute using a NanoAssemblr microfluidic mixer (Precision Nanosystems). NLuc LNPs were formulated at an mRNA concentration of 15 ng/μL for in vitro and in vivo experiments and 100 ng/μL for TEM and NGI experiments with an *N*/*P* ratio of 5.67. Cre B‐1 was formulated at a concentration of 150 ng/uL with an *N*/*P* ratio of 5.67. After formulation, LNPs were dialyzed against 1× PBS pH = 7.4 with >500× sample volume overnight using Slide‐A‐Lyzer™ G2 Dialysis Cassettes with a 10‐kDa molecular weight cutoff (87730, Thermo Fisher Scientific). Formulations were stored at 4°C until use.

### 5.4 Aerosol generation

Each formulation was aerosolized using an Aerogen Solo vibrating mesh nebulizer (Aerogen Ltd.). After aerosol generation, formulations were collected in a 1.5‐mL Eppendorf tube for use.

### 5.5 LNP characterization

Size, PDI, and zeta potential of LNPs were measured using Zetasizer Nano‐ZS (Malvern Instruments). All samples were diluted with 0.1× PBS to a final mRNA concentration of 0.75 ng/μL. Size and PDI were measured in a UV‐Cuvette micro (759200, BrandTech) and zeta potential was measured in a Folded Capillary Zeta Cell (DTS1070, Malvern).

Encapsulation efficiency was evaluated by the Quant‐IT RiboGreen RNA Assay Kit (R11490, Thermo Fisher Scientific). LNP samples were prepared to reach a final concentration of 0.6 ng/μL in 1× Tris‐EDTA (TE) to measure unencapsulated mRNA and 1% Triton to measure total mRNA. The high‐range standard curve was prepared with ribosomal RNA from the kit in both 1× TE and 1% Triton. Both samples and standards were added at a volume of 100 μL to a 96‐well black clear bottom plate (3631, Corning). After a 10‐min incubation at 37°C, 100 μL of RiboGreen RNA Reagent diluted 200‐fold was added to each well. Sample fluorescence was then measured with a SpectraMax M3 plate reader (Molecular Devices) at 480 nm excitation and 520 nm emission. Encapsulation efficiency was calculated with the following equation: (1−[unencapsulated mRNA]/[total mRNA])*100.

### 5.6 ALI cell culture

Calu‐3 cells (HTB‐55, American Type Culture Collection) from passages 12 to 15 were grown in Eagle's minimum essential media supplemented with 10% fetal bovine serum, 0.1 mM nonessential amino acids, 1 mM sodium pyruvate, and 1× penicillin/streptomycin. After reaching ~80% confluency in a flask, the cells were passaged and seeded at a density of 300,000 cells/cm^2^ in 200 μL on the apical side of ThinCert™ CellCoat™ 24‐Well Cell Culture Inserts (662641, Greiner Bio‐One). After 3 days, media on the apical side were removed and the cells were cultured at ALI. The cells were incubated at 37°C in a 5% CO_2_ atmosphere, and media on the basolateral side were refreshed every 2–3 days.

TEER values were measured using a Millicell ERS‐2 with silver/silver chloride electrodes (MERS00002, Millipore Sigma). Transwells were incubated in HBSS on both the apical and basolateral sides for 15 min at 37°C in a 5% CO_2_ atmosphere before TEER measurements were taken. Values were calculated by subtracting the resistance of the blank Transwell and then multiplying by the surface area. Cells were used for experiments after 1 week of ALI culture with TEER values >400 Ω*cm^2^.

### 5.7 In vitro transfection efficiency with ALI Calu‐3 cells

Transfection efficiency of LNPs was assessed in ALI Calu‐3 cells by adding 1000 ng to the apical side of the ALI Calu‐3 cells in 100 μL final volume. Cells were harvested 24 h later by scraping each Transwell and transferring with a multichannel to a white plate containing 100 μL of furimazine from the Nano‐Glo Luciferase Assay System (N1110, Promega). Samples were incubated for 3 min at room temperature before analysis with a SpectraMax M3 plate reader (Molecular Devices).

### 5.8 In vivo transfection efficiency with BALB/c mice

Animal protocols were approved by the Institutional Animal Care and Use Committee (IACUC; AUP‐2023‐00049) at the University of Texas at Austin. BALB/c mice (female, 6–8 weeks) were purchased from Charles River. Of note, there are no differences in basal lung function parameters such as breathing frequency and tidal volume between male and female mice. As a result, since our studies here used healthy mice, there are no expected differences.^67^ Mice were acclimated for at least 1 week before the study.

Mice were dosed intratracheally with LNPs delivering 750 ng NLuc mRNA in approximately 50 μL while anesthetized under a continuous flow of 2% isoflurane. After 24 h, mice were sacrificed, and the lungs were harvested and separated into the five lobes (left, cranial, middle, caudal, and accessory). Lobes were submerged in 400 μL of substrate from the Nano‐Glo Luciferase Assay System for 5 min. Luminescence was measured with In Vivo Imaging System (IVIS) with an exposure time of 1 s, medium binning, and an F‐stop of 1 and quantified with Living Image (PerkinElmer).

### 5.9 In vivo delivery with Ai9 mice

Ai9 mice (female, 6–8 weeks) were purchased from Jackson (007909). Mice were acclimated for at least 1 week before the study. Mice were dosed intratracheally with LNPs delivering 0.5 mg/kg Cre mRNA (L‐7211, TriLink) in approximately 50 μL while anesthetized under a continuous flow of 2% isoflurane. Mice were dosed once every other day over a period of 4 days. Three days after the last dose, the lungs were harvested.

To visualize the fluorescence of tdTomato, the lungs were imaged with IVIS at an exposure time of 5 s with 535 nm excitation and 580 nm emission, medium binning, and an F‐stop of 1.

To prepare the lung cells for single‐cell suspension, the tissue was finely minced and incubated for 1 h at 37°C in digestion medium consisting of 90 units/mL Collagenase Type I (SCR103, Millipore Sigma), 50 units/mL DNase I (11284932001, Sigma‐Aldrich), and 60 units/mL Hyalurinodase (H3506, Sigma‐Aldrich) in DMEM as previously described.^31^ After incubation, the digestion medium was quenched with DMEM +20% FBS, and tissues were filtered through a 70‐μm filter. Cells were washed once with 1× PBS before ACK lysis with 5 mL ACK Lysing Buffer (A1049201, Thermo Fisher Scientific) for 3 min at room temperature. Cells were washed in 1× PBS and resuspended in 50 μL 1× PBS for staining.

Each sample was stained with 1 μL of 1:10 dilution of Zombie NIR (423105, BioLegend) and incubated at 4°C for 30 min. Cells were then washed with 1 mL of 1× PBS twice, resuspended in 50 μL 1× PBS + 2% FBS + 0.05% sodium azide, and then stained with 1 μL of each of the following antibodies: Pacific Blue anti‐mouse CD45 (103126, BioLegend), Alexa Fluor 488 anti‐mouse CD31 (102414, BioLegend), and Alexa Fluor 647 anti‐mouse CD326 (Ep‐CAM) (118212, BioLegend). Cells were analyzed using the Attune NxT Flow Cytometer.

### 5.10 Next Generation Impactor

Aerosol distributions of LNPs were assessed through NGI (MSP Corporation). A T‐piece plug (device) was used to connect the Aerogen Solo vibrating mesh nebulizer to the induction port. Before each run, all NGI components were transferred to a cold room to prechill to 4°C for at least 90 min.^68^ LNPs were loaded into the nebulizer at 1 mL per formulation. The flow rate was operated at 15 L/min. The cutoff diameters for each stage at this flow rate are 14.1 μm for stage 1, 8.61 μm for stage 2, 5.39 μm for Stage 3, 3.30 μm for stage 4, 2.08 μm for stage 5, 1.36 μm for stage 6, and 0.98 μm for stage 7.^58^, ^69^

The LNPs were collected by first wrapping the induction port and device with parafilm. Then, 3 mL of 1× TE was added to these components as well as stages 1–7 and the MOC. The buffer was swirled around each component ~10 times. After collection, the amount of mRNA in each stage was determined with the RiboGreen assay.

### 5.11 Calculation of EF, FPF_5μm_ , MMAD, and GSD

The EF was calculated from the total mass collected from the stages divided by the total mass emitted from the device. FPF_5μm_ was interpolated from the graph of cumulative percentage of emitted dose versus particle cutoff size. The MMAD was calculated by plotting the log cumulative fraction of drug versus the aerodynamic diameter to determine the diameter at which 50% of particles were smaller or larger. The GSD was calculated with the aerodynamic diameters corresponding to 15.87% and 84.13% determined by plotting the cumulative percentage of mass less than the stated aerodynamic diameter versus aerodynamic diameter (log).

### 5.12 Transmission electron microscopy

LNP structures were visualized using a FEI Tecnai TEM. For each sample, 5 μL of LNP were loaded onto a Carbon Type‐A 300 mesh Copper grid (01820, Ted Pella). After incubation for 5 min, the sample was blotted off with filter paper. The grid was then washed with 5 μL of H_2_O. Negative staining was performed by adding 5 μL of 2% uranyl acetate. Imaging was performed at 43,000× magnification while operating at 80 kV.

### 5.13 Statistical analysis

All *p* values were calculated using a Student's *t* test assuming equal SDs and a Gaussian distribution; *p < 0.05, **p < 0.01, ***p < 0.001, and ****p < 0.0001 were considered statistically significant. Data values were presented as mean ± SD. All analysis was done in GraphPad Prism (version 8.4.3).

## AUTHOR CONTRIBUTIONS

**Mae M. Lewis:** Conceptualization (equal); formal analysis (lead); investigation (lead); methodology (lead); writing – original draft (lead). **Melissa R. Soto:** Investigation (supporting); methodology (supporting); writing – review and editing (supporting). **Esther Y. Maier:** Investigation (supporting); methodology (supporting). **Steven D. Wulfe:** Investigation (supporting); writing – review and editing (supporting). **Sandy Bakheet:** Investigation (supporting). **Hannah Obregon:** Investigation (supporting). **Debadyuti Ghosh:** Conceptualization (equal); funding acquisition (lead); writing – review and editing (supporting).

## CONFLICT OF INTEREST STATEMENT

Dedadyuti Ghosh and Mae M. Lewis have filed a provisional patent on this work.

### PEER REVIEW

The peer review history for this article is available at https://www.webofscience.com/api/gateway/wos/peer-review/10.1002/btm2.10580.

## Supporting information


**FIGURE S1.** In vitro delivery of nonaerosolized LNPs in ALI Calu‐3 cells. Quantification of luminescence from ALI Calu‐3 cells 24 h after transfection with nonaerosolized (a) Set 1 and (b) Set 2 LNPs delivering 1000 ng NLuc mRNA (*n* = 3; mean ± standard deviation).
**FIGURE S2.** Physicochemical properties. (a) Size (nm) by DLS (*n* = 3; mean ± standard deviation [SD]). (b) PDI by DLS (*n* = 3; mean ± SD). (c) Zeta potential (mV) by DLS (*n* = 3; mean ± SD). (d) Encapsulation efficiency (%) by RiboGreen (*n* = 2; mean ± SD).
**FIGURE S3.** Quantification of radiance in each individual lung lobe from Figure 2b.

## Data Availability

The data that support the findings of this study are available from the corresponding author upon reasonable request.
