# Supplementary material for: Optimization of ionizable lipids for aerosolizable mRNA lipid nanoparticles
Source: Bioeng Transl Med. 2023 Aug 21;8(6):e10580. doi: 10.1002/btm2.10580 (PMC10658486; doi:10.1002/btm2.10580)
Supplement: Supplementary file 1 — FIGURE S1. In vitro delivery of nonaerosolized LNPs in ALI Calu‐3 cells. Quantification of luminescence from ALI Calu‐3 cells 24 h after transfection with nonaerosolized (a) Set 1 and (b) Set 2 LNPs delivering 1000 ng NLuc mRNA (n = 3; mean ± standard deviation). FIGURE S2. Physicochemical properties. (a) Size (nm) by DLS (n = 3; mean ± standard deviation [SD]). (b) PDI by DLS (n = 3; mean ± SD). (c) Zeta potential (mV) by DLS (n = 3; mean ± SD). (d) Encapsulation efficiency (%) by RiboGreen (n = 2; mean ± SD). FIGURE S3. Quantification of radiance in each individual lung lobe from Figure 2b. [file BTM2-8-e10580-s001.docx]

**Supporting Information**

**Optimization of ionizable lipids for aerosolizable mRNA lipid nanoparticles**

Mae M. Lewis ^1^, Melissa R. Soto ^2^, Esther Y. Maier ^3^, Steven D. Wulfe ^2^, Sandy Bakheet ^2^, Hannah Obregon ^2^, Debadyuti Ghosh ^2, †^

^1^ Department of Biomedical Engineering, The University of Texas at Austin, Austin, Texas, USA

^2^ Division of Molecular Pharmaceutics and Drug Delivery, College of Pharmacy, The University of Texas at Austin, Austin, Texas, USA

^3^ Drug Dynamics Institute, The University of Texas at Austin, Austin, Texas, USA

^†^ Corresponding Author

Corresponding Author email address: dghosh@austin.utexas.edu

Corresponding Author Address: Division of Molecular Pharmaceutics and Drug Delivery, College of Pharmacy, The University of Texas at Austin, 2409 University Ave, Austin, TX 78712, USA

**This file includes:**

Figure S1. In vitro delivery of non-aerosolized LNPs in ALI Calu-3 cells.

Figure S2. Physicochemical properties.

Figure S3. Quantification of radiance in each individual lung lobe from Figure 2b.

| a  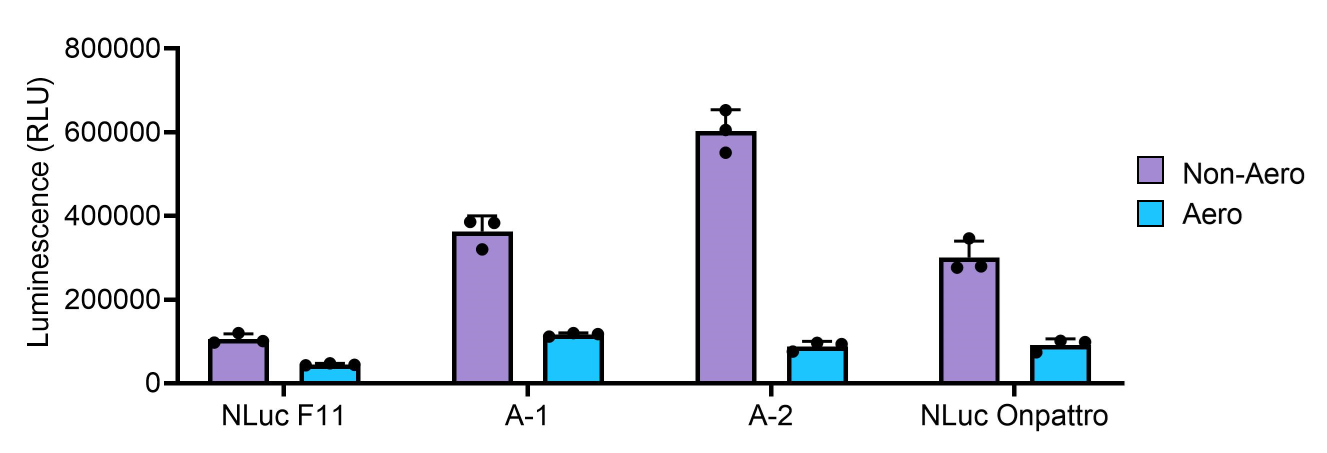 |
| --- |
| b  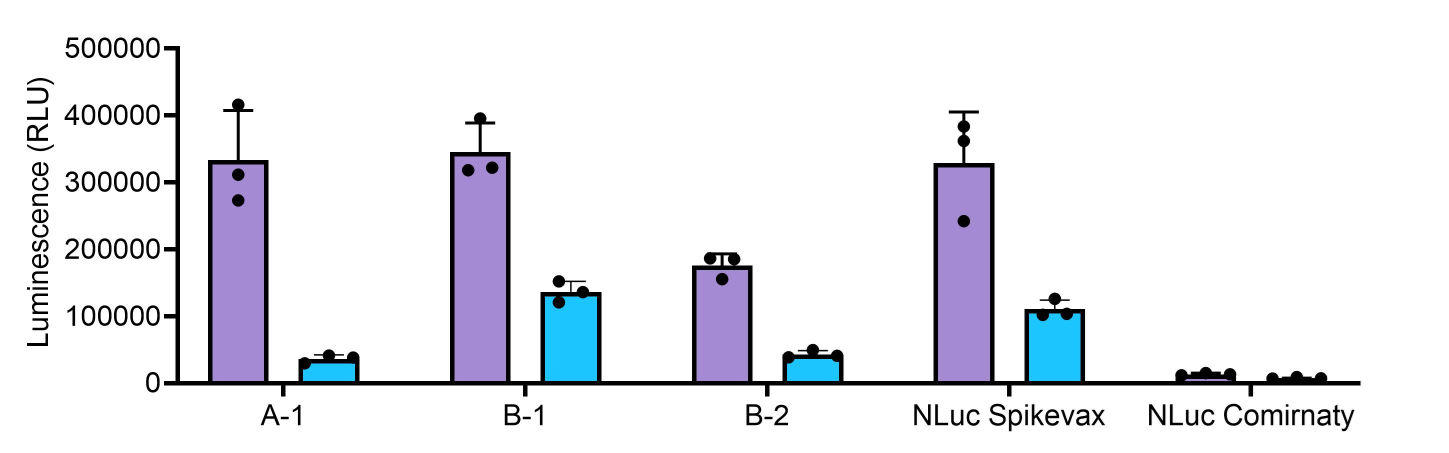 |

**Figure S1. In vitro delivery of non-aerosolized LNPs in ALI Calu-3 cells.** Quantification of luminescence from ALI Calu-3 cells 24 h after transfection with non-aerosolized **a)** Set 1 and **b)** Set 2 LNPs delivering 1000 ng NLuc mRNA (*n* = 3; mean ± standard deviation).

| 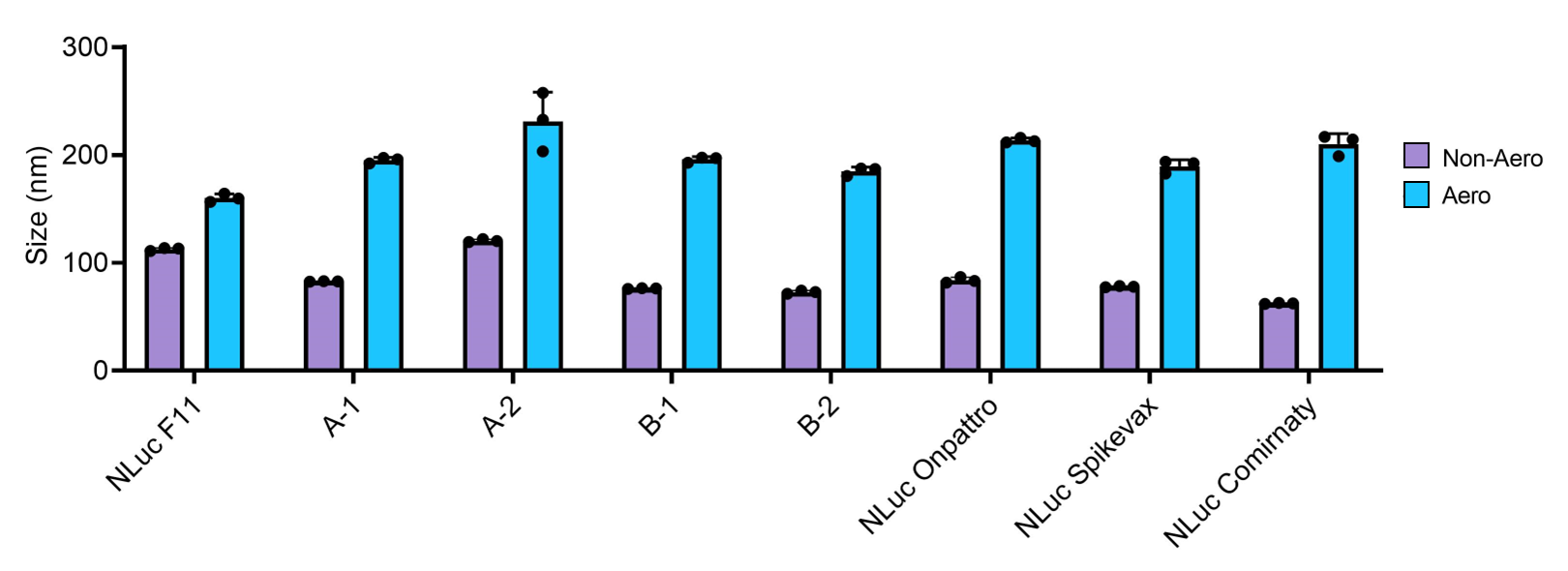a |
| --- |
| b  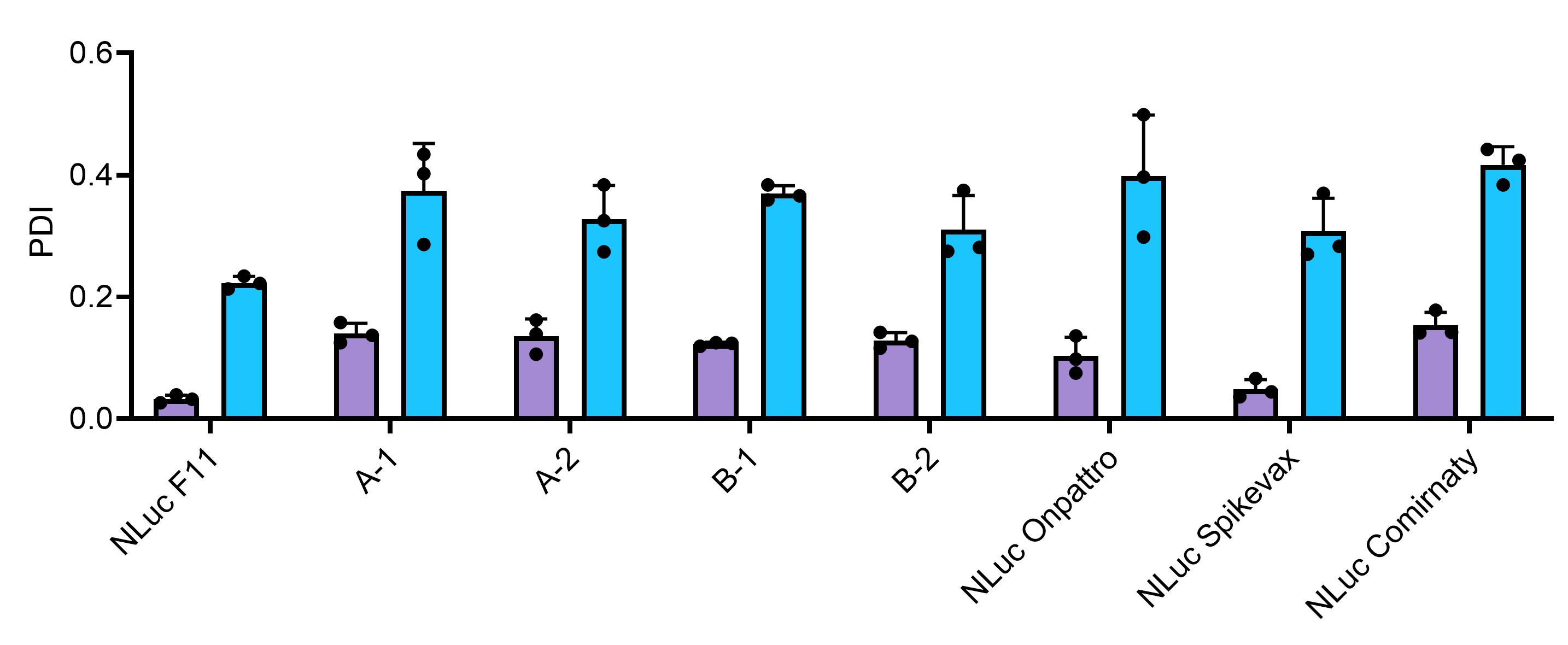 |
| c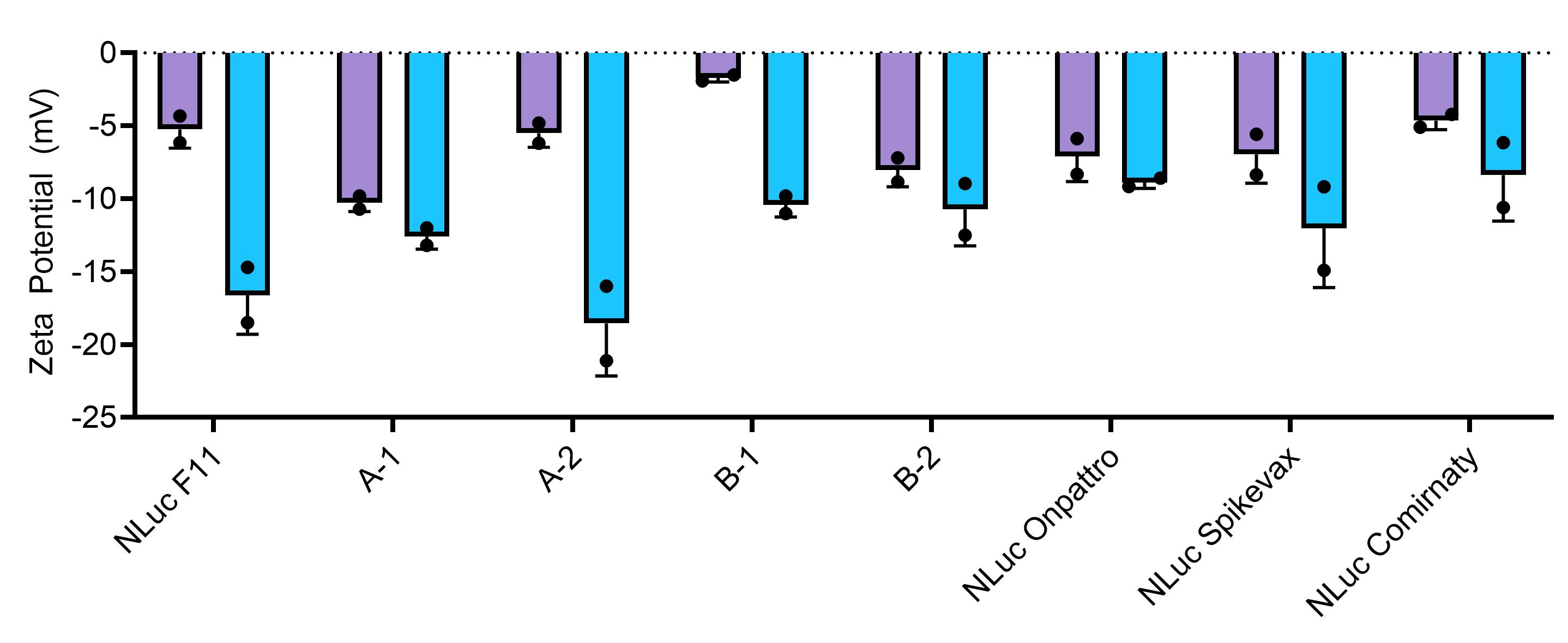 |
| d  **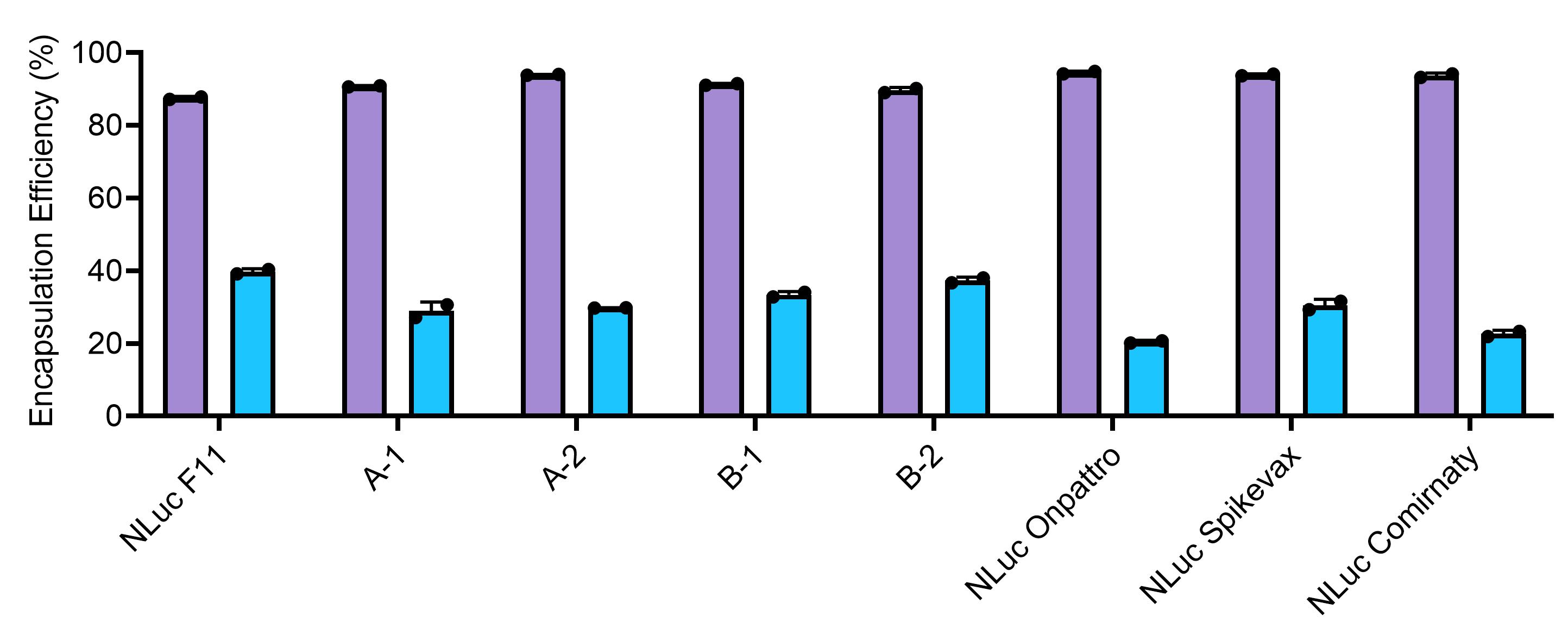** |

**Figure S2. Physicochemical properties. a)** Size (nm) by DLS (*n* = 3; mean ± standard deviation [SD]). **b)** PDI by DLS (*n* = 3; mean ± SD). **c)** Zeta potential (mV) by DLS (*n* = 3; mean ± SD). **d)** Encapsulation efficiency (%) by RiboGreen (*n* = 2; mean ± SD).


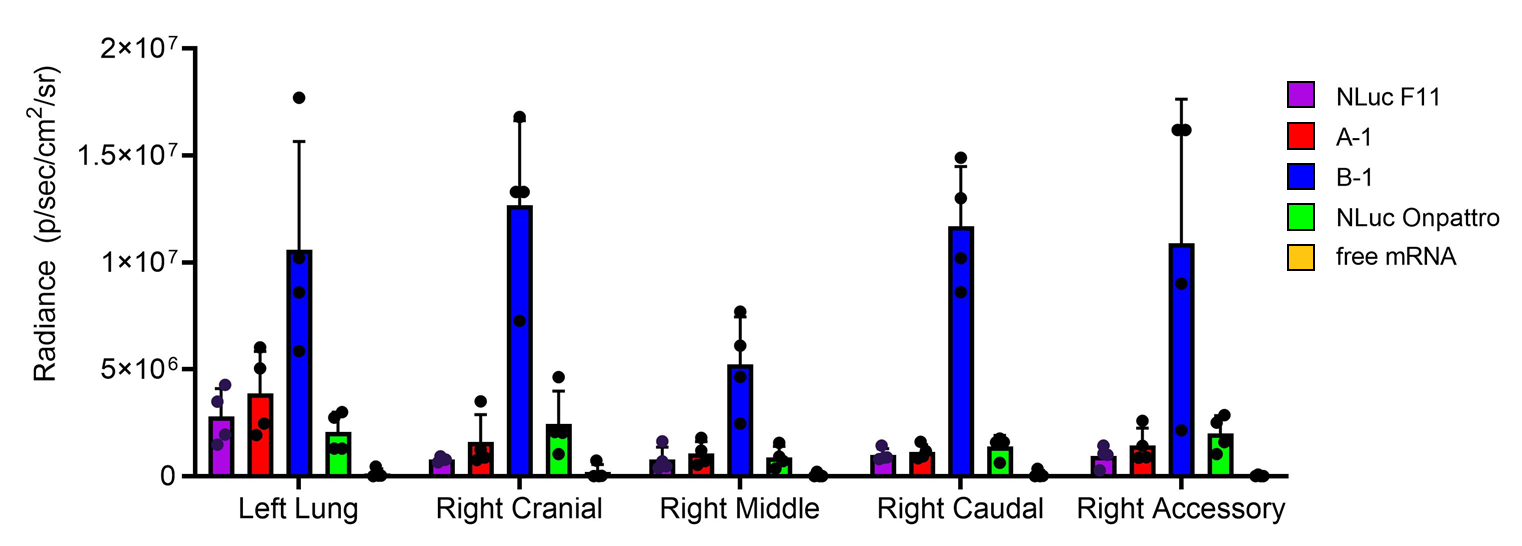


**Figure S3. Quantification of radiance in each individual lung lobe from Figure 2b.**
